# The Year That Has Passed Us By: Animals in Our Life of COVID-19

**DOI:** 10.3390/ani11020395

**Published:** 2021-02-04

**Authors:** Aubrey H. Fine

**Affiliations:** Department of Education, California State Poly University, Pomona, CA 91768, USA; ahfine@cpp.edu

## 1. Introduction

The year 2020 has been a dreadful one: a living nightmare that has changed and impacted the lives of many. On 11 March 2020, the World Health Organization announced that COVID-19 was to be considered a worldwide pandemic. Not since the Spanish flu of the last century have citizens of the world been threatened with such global devastation and catastrophe. Without exception, the pandemic has commanded the attention of the entire world. As of this writing, there are over 450,000 deaths in the US alone, and although vaccines are now being dispensed, it is likely that many more will die because of this horrific deadly disease. Perhaps the most significant and overshadowing effect of the pandemic has been the growing confinement of individual humans.

Christakis in his book Apollo’s Arrow discusses his belief that one of the major tragedies of humanity has been our struggle to learn from the past. It is apparent that different generations have suffered from various types of pandemics or tragic events, but unfortunately, humans tend to forget easily when the issues are abated, and we try to pick up our lives and move forward [1]. Christakis believes that these are missed opportunities to learn from our lessons. I believe that we are experiencing another wake-up call with regard to learning from our past. Hopefully, we can learn from our history and put into place precautionary measures that will help humanity to persevere. Learning from the past should help us to recognize that there are options that can be implemented to foster resilience in the face of catastrophes.

The intention of this Special Issue of Animals is to address some of the psychosocial stressors that many across the globe have experienced over the past year and to highlight the transforming roles of animal companions during this period. As one can only imagine, people are turning to their pets and other outlets to release their stresses associated with being confined. Animals represent a positive outlet to release some of the tensions and provide a healthy outlet for engagement. It is also the intention of the articles within this issue to provide an overview of the current state of research being conducted on these roles and the roles that animals are taking on during the pandemic.

## 2. The Roles of Animals Acting as Social Support

The scientific community has earnestly been interested in understanding and providing solutions to support people who are living through this personal nightmare. We have all witnessed the tragic impact that COVID-19 is having. Unlike hurricanes that leave physical devastation and destruction after they hit landfall, COVID-19 has left a trail of physically and mentally vulnerable people who feel alone and afraid. Some have developed coping strategies that have eased their distress during this period of time. These strategies have acted as emotional and social supports helping them to get by. 

The major impetus of this volume is to investigate the changing roles of animals during the pandemic and to get a clearer understanding of their impact. Fine pointed out that the literature is now filled with studies highlighting how interacting with animals has helped people to feel less stress and anxiety and has supported some people in reducing feelings of loneliness and isolation [2]. Today, there are numerous accepted theoretical models that explain our desire to interact with animals, including the attachment theory, the biophilia hypothesis, and the social support theory [2]. The social support theory appears the most relevant in conceptualizing the value of animal companionships during the pandemic. 

It is now more commonly accepted that animals have been recognized as providing social support through relationships with them [2]. As we have witnessed during the pandemic, our pets tend to be more available compared to our human friends, which may be one reason we have witnessed an increase in people adopting pets from shelters [3]. Additionally, many individuals, young and old, seek the comfort of their companion animals when they are feeling distress due to the relationships they have or to the lack of them [4]. Furthermore, this belief holds true for various populations, such as the elderly and people with chronic health problems and disabilities [5].

Scientists have begun investigating the changes we are witnessing in the ways that people interact with their pets as a result of being sequestered. A majority of the studies seem to indicate that meaningful companionship with animals could result in an increase in resilience in combating isolation. Ratschen et al. suggested that pets during the pandemic acted as a critical source of emotional support during lockdown (without regard to the actual species) [6]. Furthermore, Olivia and Johnston hypothesized that pets might have a healthy impact on their humans in enhancing their state of relaxation [7]. Daily positive interactions with pets provided opportunities to play and interact more normally. Normality is a variable that has to be considered. Pets can help humans to institute a daily routine that can be enriching and restoring. For many, interacting with animals may [2]. Research in the papers reviewed seemed to point to the value of companionship. The literature is filled with research demonstrating that companionship with animals is one of the greatest outcomes derived from human–animal interactions [2,8]. In essence, having pets such as dogs provides opportunities to maintain a routine that includes leaving the house to take short walks with one’s animal. Physical connections were also highlighted as a benefit of having dogs, while cat owners suggested that being around their cat supported their mood state [7]. Young et al’s research highlighted the importance of physical contact and touch as a result of human–animal interactions [9]. Many of their subjects pointed out that being able to pet and touch their pets was extremely valuable in reducing their stress during the pandemic. 

Finally, Hoy-Gerlach et al. pointed out our interactions with pets could also be valuable in enhancing opportunities for physical exercise and activity [10]. Research on dog walking has pointed out that it is a valuable motivator to support adherence to exercise and can foster good physical and mental health [11]. Particularly, during the pandemic, being able to walk outdoors may be extremely helpful, especially when done safely.

Although a majority of the early papers published on this topic highlighted the benefits of pets, they also pointed out some of the challenges people are experiencing in keeping or adopting new pets during the pandemic. Numerous commentaries suggested several issues including the economic and care burdens experienced by families taking care of their pets, the identified changes in their daily routines (including pets) as a result of being sequestered, and the healthcare concerns of the pets [12]. Nevertheless, throughout the globe, agencies are establishing food banks for the growing number of people who cannot afford pet food and supplies as well as arranging for foster care in the event of illness or inability to take care of their animals [10]. 

It is incumbent for researchers to also study the impact on pets who are now spending much more time with their families (for some a blessing in disguise). From the pets’ perspective, the disruption to their daily lives may cause some undue stress because of the confinement and changes in their routine. Likewise, it must be assumed that return to post-pandemic era will cause stress and anxiety as pets adjust to the “new normal” that no longer includes undivided attention.

## 3. Conclusions

This pandemic has motivated many researchers and scientists to reconsider and expand upon some of the psychosocial options presently made available to support global citizens in achieving more optimal living. The purpose of the Preface was to explore the current landscape of what we presently know about the hardships that the pandemic has caused as well as our present understanding of the changing roles of companion animals during this period. As the pandemic continues to spread and has a tremendous impact on the physical and social lives of citizens globally, the scientific community must contribute to the body of knowledge, providing tangible options and policies that will help our communities and their citizens to recover. I urge and challenge the scientific community to vigilantly continue their collective efforts to study the changing roles of animals during this period, not only from a human perspective but also from the animals’ point of view. We need to appreciate that these findings will not only be helpful in dealing with this present pandemic, but also in providing applicable information to be applied in future global catastrophes. As noted earlier in Christakis’s book Apollo’s Arrow, we need as a society to look back at our lessons in dealing with catastrophic events and come up with plans that are based on the knowledge we have gained from them.

Above all, scientific clarity is needed to understand how the pandemic has impacted the lives of our companion animals as well as the animals’ contributions to human lifestyles. More attention should be given to answering the many questions we are now considering, including understanding why animals seem to be more supportive to populations that are more vulnerable to mental health challenges, as well as clarifying the impact on people in various developmental stages of life. Additionally, research is still needed to better conceptualize how confinement and changes in daily life impact our pets and what options should be put into place to safeguard their wellbeing. Einstein once said, “the world as we have created it is a process of our thinking. It cannot be changed without changing our thinking.” We must begin to expand the way we think and react to disasters as significant as the COVID-19 pandemic. By taking steps that are advantageous, we may be able to come up with solutions that will have a stronger impact on the devastation that we are witnessing and living through at this time.
